# Factors associated with the timing of the first prenatal ultrasound in Canada

**DOI:** 10.1186/s12884-019-2309-4

**Published:** 2019-05-10

**Authors:** Peri Abdullah, Christine Kurtz Landy, Hugh McCague, Alison Macpherson, Hala Tamim

**Affiliations:** 1 Kinesiology and Health Science, York University, 4700 Keele Street, Toronto, Ontario M3J 1P3 Canada; 2 Health, Nursing and Environmental Studies, York University, 4700 Keele Street, Toronto, Ontario M3J 1P3 Canada; 3 Institute for Social Research, York University, 4700 Keele Street, Toronto, Ontario M3J 1P3 Canada

**Keywords:** Prenatal ultrasound, Canada, Epidemiology, Multinomial logistic regression

## Abstract

**Background:**

The aim of this study was to investigate the factors associated with the timing of the first prenatal ultrasound in Canada.

**Methods:**

This was a secondary data analysis of the Maternity Experiences Survey, a cross-sectional survey covering different aspects of pregnancy, labour, birth and the post-partum period. Bivariate and multivariate multinomial logistic regressions were performed to assess the relationship between timing of first prenatal ultrasound and different independent variables.

**Results:**

68.4% of Canadian women received an optimally timed first prenatal ultrasound, 27.4% received early ultrasounds and 4.3% received late ultrasound. The highest prevalence of early ultrasound was in Ontario (33.3%) and the lowest was in Manitoba (13.3%). The highest prevalence of late ultrasound was found in Manitoba (12.1%) and the lowest was in British Columbia and Ontario (3.5% each). The highest prevalence of optimal timing of first prenatal ultrasound was in Quebec (77%) and the lowest was in Ontario (63.2%). Factors influencing the timing of ultrasound included: *Early –* maternal age < 20 (adjusted OR = 0.54, 95%CI:0.34–0.84), alcohol use during pregnancy (adjusted OR = 0.69, 95%CI:0.53–0.90), history of premature birth (adjusted OR = 1.41, 95%CI:1.06–1.89), multiparity (adjusted OR = 0.67, 95%CI:0.57–0.78), born outside of Canada (adjusted OR = 0.82, 95%CI:0.67–0.99), prenatal care in Newfoundland and Labrador (adjusted OR = 1.66, 95%CI:1.20–1.30), Nova Scotia (adjusted OR = 1.68, 95%CI:1.25–2.28), Ontario (adjusted OR = 2.16, 95%CI:1.76–2.65), Saskatchewan (adjusted OR = 1.50, 95%CI:1.05–2.14), Alberta (adjusted OR = 1.37, 95%CI:1.05–1.77) British Columbia (adjusted OR = 1.90, 95%CI:1.45–2.50) and Manitoba (adjusted OR = 0.66, 95%CI:0.45–0.98) *Late –* unintended pregnancy (adjusted OR = 1.89, 95%CI:1.38–2.59), born outside of Canada (adjusted OR = 1.75, 95%CI:1.14–2.68), prenatal care in Manitoba (adjusted OR = 2.88, 95%CI:1.64–5.05) and the Territories (adjusted OR = 4.50, 95%CI:2.27–8.93). An interaction between history of miscarriage and having ‘other’ prenatal care provider significantly affected timing of ultrasound (adjusted OR = 0.31, 95%CI:0.14–0.66).

**Conclusion:**

Only 68% of Canadian women received an optimally timed prenatal ultrasound which was influenced by several factors including province of prenatal care, maternal age and country of birth, and an interaction effect between prenatal care provider and history of miscarriage. These findings establish a baseline of factors influencing the timing of prenatal ultrasound in Canada, which can be built upon by future studies.

## Background

Ultrasound has been used in obstetrics since the 1950s and has become an essential part of current prenatal care [1]. The use of ultrasound in pregnancy can provide valuable information that can predict fetal outcomes and aid in the detection of cardiac [2], gastrointestinal [3], renal [4], and neural abnormalities [5] as well as chromosomal anomalies including Down’s Syndrome [1]. Routine ultrasound in pregnancy is also useful for determination of multiple pregnancies and gestational age, and may be associated with a lower likelihood of inductions after 42 weeks [6]. Current recommendations by the Society of Obstetricians and Gynecologists of Canada (SOGC) state that all pregnant women should be offered an ultrasound scan between 18 and 22 weeks to screen for fetal anomalies and to provide information about the placenta, gestational age and number of fetuses [7]. In addition, the SOGC recommends that all pregnant patients be offered an ultrasound scan between 11 and 14 weeks of pregnancy to confirm gestational age and viability as well as investigate the number of fetuses, early anatomical assessment and nuchal translucency [8].

Ultrasound can be used to detect a pregnancy as early as 5 weeks [9]. In early pregnancy, ultrasound is indicated for conditions such as: therapeutic abortions, threatened miscarriages and their complications, uncertain menstrual dates, twin pregnancies, abnormal pregnancies (e.g. ectopic, molar etc.) and pelvic masses [10]. However, since the early stages of pregnancy are so sensitive to any external changes [11], early prenatal ultrasound should be used with caution. In fact, using transvaginal ultrasound in early pregnancy has been associated with increased cell death rates in the developing human fetus [12]. Similarly, the use of Doppler ultrasound in early pregnancy has been associated with cell death in the liver of the rat fetus [13]. In addition, having an ultrasound too early might lead to misinterpretation and, consequently, unnecessary interventions that can harm an otherwise normal pregnancy [14].

Similarly, having the first prenatal ultrasound later in pregnancy can have a different set of consequences. First, the estimation of the gestational age of the fetus becomes increasingly less reliable as the pregnancy progresses [15, 16], reaching a margin of error of more than 20 days in the third trimester [15]. This can have consequences when dealing with many situations including preterm labour and intrauterine growth restriction [15]. Moreover, generally speaking, presenting to obstetric care later in pregnancy can lead to adverse outcomes such as low birth weight, infant and neonatal mortality [17] and congenital malformations [18]. Finally, late access to prenatal care can result in missed opportunities of timely screening using diagnostic tests such as prenatal ultrasound [19].

Women who attend prenatal care later in pregnancy (after the first trimester) might miss the window of an optimally timed ultrasound. Differences in those who attend prenatal care late from those who attend early have been reported previously. It has been shown that late attenders (first prenatal visit after the first trimester) tend to be teenagers, unmarried, have had multiple pregnancies [20, 21], non-European, have lower education and lower socio-economic status [21]. In Canada, inadequate prenatal care (prenatal care initiated after the 4th month of pregnancy, and having fewer prenatal care visits) was associated with being an immigrant, primiparity, smoking and alcohol use during pregnancy, and having a family doctor as the prenatal care provider [22]. In Manitoba, inadequate prenatal care (prenatal care between month 1 and 6 and having fewer than 8 prenatal care visits) was associated with young maternal age, being a single parent, having had 4 or more births and lower income [23].

Canada is the second largest country in the world by total area [24], however, it is sparsely populated with a total population of 37,314,442 [25]. Canada consists of 10 provinces and three territories. Approximately 86% of the population resides in four provinces, namely Quebec and Ontario in the east and Alberta and British Columbia in the west [25]. Average income is fairly equal across the provinces [26].The territories tend to be underserviced [27] with a lower socioeconomic status [28]. Canada has a publicly funded health care system. This means that obstetric care, including prenatal ultrasounds is publicly funded [29]. No studies have been found that addressed the factors associated with the timing of the first prenatal ultrasound in Canada or elsewhere. This is an important area to investigate because it can be used to better focus educational efforts and interventional efforts aimed at the optimization of prenatal ultrasound utilization in Canada. Moreover, findings from such an investigation may be used to help address issues such as over- and underutilization of prenatal ultrasound. The aim of this study is to investigate the factors associated with the timing of the first prenatal ultrasound in Canada using a national database, the Maternity Experiences Survey (MES).

## Methods

This study was a secondary data analysis of the Maternity Experiences Survey (MES), a cross-sectional survey conducted following the 2006 Canadian Census of Population. The MES was the first national survey in Canada devoted to women’s experiences of pregnancy, labour, birth and the postpartum period. The objective of this survey was to collect data from new mothers on perinatal health indicators such as: maternal health, prenatal care, labour and delivery, newborn health, breastfeeding, postpartum care, sources of information during pregnancy as well as overall experience. The MES was an initiative of the Canadian Perinatal Surveillance System of the Public Health Agency of Canada [30].

The target population for this survey was women who had given birth to a single baby in Canada between February 15 and May 15 of 2006 in Canada’s 10 provinces, or between November 1, 2005 and February 1, 2006 in Canada’s 3 territories. Participants were at least 15 years of age at the time of giving birth and had to have their baby spend at least one night per month with them. Exclusion criteria included women who lived in collective dwellings or on First Nations reserves. The final sample included 6421 women who had completed the survey and had given Statistics Canada consent to share their responses with the sponsor (Public Health Agency of Canada – Canadian Perinatal Surveillance System). Participation in the survey was voluntary with a response rate of 78%. In the provinces, data collection was conducted using a Computer Assisted Telephone Interview (CATI). In the territories, paper versions of the questionnaire were filled out during a personal interview if performing a CATI was not possible. The MES protocol has been reviewed by the Health Canada’s Science Advisory Board and Research Ethics Board and the Federal Privacy Commissioner, and approved by the Statistics Canada’s Policy Committee. Since this project was based on secondary data analysis of the MES, institutional ethics approval was not required. Detailed methodology of the MES has been described previously [30].

For this study, respondents who received prenatal care outside of Canada accounted for about 0.01% of the sample and were excluded. The outcome variable was ‘timing of first ultrasound’ and had three levels: early (defined as receiving the first prenatal ultrasound before 11 weeks of pregnancy), optimal (defined as receiving the first prenatal ultrasound between 11 and 22 weeks of pregnancy) and late (defined as receiving the first prenatal ultrasound after 22 weeks of pregnancy). These categories were chosen based on the SOGC recommendations that were in place at the time of the survey. In 2007, the SOGC recommendations for fetal aneuploidy stated that all pregnant women be offered prenatal screening test for fetal aneuploidy, some of which included a prenatal ultrasound between 11 and 14 weeks [31]. Similarly, this ultrasound was also recommended by the SOGC in 2003 to be offered as part of a comprehensive prenatal screening program [10]. The SOGC also recommended in 1999 that all pregnant patients be offered an ultrasound around 18–19 weeks to screen for structural anomalies [32]. However, in 2009 the SOGC recommended this ultrasound at 18–22 weeks [33]. In the present study the cut-off for the ‘late’ category was 22 weeks, which is in keeping with the recommendations that are closest to the time when the data were collected as well as current recommendations.

Information about this variable was collected from responses to the question “How many weeks pregnant were you when you had your first ultrasound?”. The covariates assessed were grouped into several categories, the first of which is maternal factors which included: using fertility medications or procedures to get pregnant with the index pregnancy, health problems before pregnancy that warrant additional care during the index pregnancy, health problems during the index pregnancy that warrant additional care during pregnancy, pre-pregnancy Body Mass Index (BMI) and whether the pregnancy was intended. The latter was obtained from the question “thinking back to just before you became pregnant, would you say that you wanted to be pregnant...?” with the following responses: sooner, then, later and not at all. The first 2 were combined into ‘intended’ and the second 2 were combined into ‘unintended’. The second category of covariates was behavioural risk factors and included: smoking during the last 3 months of pregnancy and alcohol use during pregnancy. The third category of covariates was reproductive history and included: parity (primiparous or multiparous), history of premature birth, history of ectopic and stillbirth (combined due to low counts), history of miscarriage and history of therapeutic abortion. The fourth category of covariates was prenatal and birth related factors and included: type of prenatal care provider, mode of delivery and birthweight. The final category of covariates was socio-demographic factors and included: maternal age, country of birth/Aboriginal status (included 3 categories: Canadian born, born outside of Canada, and Aboriginal – First Nations, Metis or Inuit), marital status (dichotomized to: with partner or with no partner), province of prenatal care, urban/rural residence, travel to another city for birth, education, employment during pregnancy and household income. The reference category for province of prenatal care was chosen to be Quebec due to its appropriate sample size and ease of interpretation of the results relative to the other provinces.

### Statistical analyses

Multinomial logistic regression is an extension of simple logistic regression [34]. Unlike simple logistic regression, multinomial logistic regression allows for the analysis of outcomes that have more than two categories [34]. For this study, Chi square tests and bivariable multinomial logistic regression models were performed to assess the relationship of the covariates with the outcome variable at the bivariate level. Multivariable multinomial logistic regression was performed to assess the relationship between the independent variables and the outcome variable (reference category: ‘optimal’) while controlling for all of the covariates. This analysis produced adjusted Odds Ratios (OR) and 95% Confidence Intervals (95%CI). The significance level was set at alpha of 0.05. In addition, several potential interactions were investigated including: a) prenatal care provider x province of prenatal care b) prenatal care provider x urban/rural c) prenatal care provider x history of miscarriage d) prenatal care provider x history of stillbirth or ectopic e) prenatal care provider x having a condition before pregnancy requiring additional care and f) prenatal care provider x having a condition during pregnancy requiring additional care. A probability survey weight and 1000 bootstrap weights were provided by Statistics Canada and applied in order to obtain results that were nationally representative. The bootstrap weights take account of the complex design of the survey and provide more accurate estimates of variance [35]. All statistical tests were performed using Stata Statistical Software version 14 (StataCorp, College Station, TX).

## Results

**Fig. 1:**
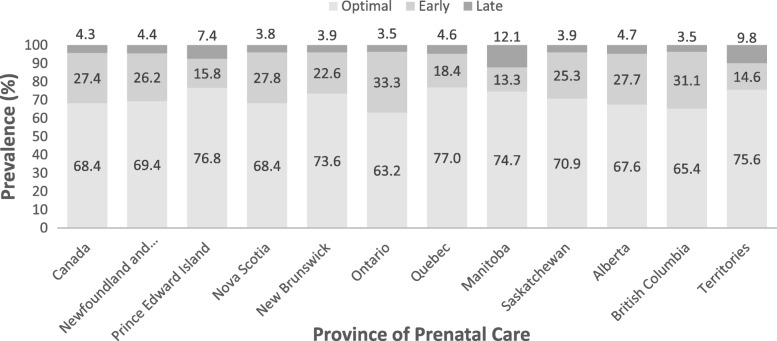
Prevalence of early, appropriate and late ultrasound in different regions of Canada. The Maternity Experiences Survey (MES)

The total weighted sample size used was around 76,000. The percentage of Canadian women receiving an optimally timed first prenatal ultrasound was 68.4%, while 27.4% of women received early ultrasounds and 4.3% received late ultrasound (Fig. 1). The province with the highest prevalence of optimal timing of first prenatal ultrasound was Quebec (77%), while the lowest prevalence of optimal timing of first prenatal ultrasound was found in Ontario (63.2%) (Fig. 1). The province with the highest prevalence of early ultrasound was Ontario (33.3%) while the lowest prevalence of early ultrasound was found in Manitoba (13.3%) (Fig. 1). Interestingly, Manitoba was also the province with the highest prevalence of late ultrasound (12.1%) (Fig. 1). The provinces with the lowest prevalence of late ultrasound were British Columbia and Ontario (3.5% each) (Fig. 1).

Table 1 shows the percentages and the unadjusted Odds Ratios (ORs) and 95% Confidence Intervals (95%CI) for timing of first prenatal ultrasound at the level of each of the covariates.

**Table 1:** Percentages and unadjusted Odds Ratios (OR) and 95% Confidence Intervals (95%CI) for timing of ultrasound at different independent variables. The Maternity Experiences Survey (MES)

Variable	Overall %	Optimal %	Early		Late	
			%	Unadjusted OR (95%CI)^a^	%	Unadjusted OR (95%CI)^a^
MATERNAL FACTORS						
Fertility medications or procedures						
Yes	4.7	42.6	55.2	3.49 (2.70–4.52)	2.2	0.81 (0.26–2.53)
No	95.3	69.8	25.9	1.00	4.4	1.00
Health problems before pregnancy						
Yes	15.3	63.8	33.7	1.40 (1.20–1.63)	2.5	0.57 (0.36–0.90)
No	84.7	69.2	26.1	1.00	4.6	1.00
Health Problems during pregnancy						
Yes	24.4	65.4	31.1	1.26 (1.15–1.44)	3.5	0.82 (0.60–1.12)
No	75.6	69.3	26.1	1.00	4.5	1.00
Pre-pregnancy BMI						
Underweight (less than 18.5)	6.1	59.3	36.5	1.63 (1.27–2.08)	4.2	1.17 (0.66–2.09)
Normal (18.5–24.99)	59.3	69.5	26.3	1.00	4.2	1.00
Overweight/obese (25+)	34.6	68.4	27.4	1.06 (0.93–1.20)	4.3	1.03 (0.77–1.36)
Intended pregnancy						
Unintended	27.1	66.7	26.3	0.98 (0.86–1.13)	7.1	2.24 (1.74–2.88)
Intended	73.0	69.1	27.7	1.00	3.3	1.00
BEHAVIOURAL RISK FACTORS						
Smoking during the last 3 months of pregnancy						
Yes	10.5	66.6	27.0	1.02 (0.84–1.23)	6.4	1.63 (1.18–2.27)
No	89.5	68.6	27.3	1.00	4.1	1.00
Alcohol during pregnancy						
Yes	10.5	75.6	20.1	0.64 (0.51–0.80)	4.3	0.88 (0.56–1.40)
No	89.6	67.6	28.1	1.00	4.3	1.00
REPRODUCTIVE HISTORY						
Parity						
Primiparous	45.5	65.7	30.8	1.00	3.5	1.00
Multiparous	54.5	70.7	24.4	0.73 (0.65–0.83)	5.0	1.32 (1.01–1.71)
History of premature birth						
Yes	5.8	64.0	32.9	1.30 (1.02–1.67)	3.1	0.80 (0.41–1.54)
No	94.2	68.8	27.1	1.00	4.2	1.00
History of ectopic pregnancy or stillbirth						
Yes	2.5	59.6	38.8	1.65 (1.18–2.32)	0.9	0.42 (0.15–1.12)
No	97.5	68.6	27.0	1.00	4.4	1.00
History of miscarriage						
Yes	22.1	60.9	36.6	1.72 (1.50–1.97)	2.5	0.61 (0.42–0.89)
No	77.9	70.5	24.7	1.00	4.8	1.00
History of therapeutic abortion						
Yes	11.8	69.4	26.3	0.94 (0.78–1.14)	4.3	0.98 (0.63–1.52)
No	88.2	68.2	27.5	1.00	4.3	1.00
PRENATAL AND BIRTH RELATED FACTORS						
Type of prenatal care provider						
OB/GYN	58.3	67.1	28.8	1.00	4.0	1.00
Family doctor/GP	34.4	70.3	25.2	0.84 (0.74–0.95)	4.5	1.07 (0.81–1.40)
Midwife and other^b^	7.3	68.5	26.1	0.89 (0.69–1.13)	5.4	1.30 (0.83–2.06)
Mode of delivery						
Vaginal	73.7	69.4	26.2	1.00	4.5	1.00
Caesarean	26.3	65.6	30.7	1.24 (1.09–1.41)	3.8	0.89 (0.66–1.21)
Birthweight						
< 2500	5.1	60.7	35.0	1.45 (1.10–1.90)	4.3	1.15 (0.61–2.14)
2500–4000	82.4	68.5	27.2	1.00	4.3	1.00
> 4000	12.6	70.6	24.8	0.89 (0.74–1.06)	4.6	1.05 (0.71–1.56)
SOCIO-DEMOGRAPHIC FACTORS						
Maternal age at delivery						
< 20	3.0	66.0	22.3	0.86 (0.62–1.19)	11.7	2.89 (1.87–4.45)
20–34	79.5	68.7	27.1	1.00	4.2	1.00
35+	17.6	67.6	29.2	1.10 (0.94–1.28)	3.2	0.76 (0.52–1.11)
Country of birth/Aboriginal status						
Born in Canada	71.8	69.1	27.4	1.00	3.6	1.00
Born outside of Canada	24.0	66.7	27.2	1.03 (0.89–1.19)	6.1	1.77 (1.34–2.34)
Aboriginal	4.2	67.8	25.8	0.96 (0.72–1.28)	6.4	1.81 (1.10–2.96)
Marital status						
With partner	91.6	68.9	27.0	0.83 (0.68–1.01)	4.1	0.60 (0.41–0.87)
No Partner	8.4	63.6	30.1	1.00	6.4	1.00
Province of prenatal care						
Quebec	23.5	77.0	18.4	1.00	4.6	1.00
Newfoundland and Labrador	1.3	69.4	26.2	1.59 (1.20–2.09)	4.4	1.05 (0.58–1.90)
Prince Edward Island	0.4	76.8	15.8	0.86 (0.65–1.14)	7.4	1.62 (1.00–2.63)
Nova Scotia	2.2	68.4	27.8	1.71 (1.33–2.18)	3.8	0.93 (0.49–1.75)
New Brunswick	1.9	73.6	22.6	1.29 (0.96–1.72)	3.9	0.88 (0.49–1.59)
Ontario	39.3	63.2	33.3	2.21 (1.86–2.61)	3.5	0.93 (0.65–1.34)
Manitoba	3.5	74.7	13.3	0.75 (0.53–1.04)	12.1	2.70 (1.77–4.10)
Saskatchewan	3.1	70.9	25.3	1.49 (1.12–1.99)	3.9	0.91 (0.48–1.72)
Alberta	12.3	67.6	27.7	1.72 (1.40–2.12)	4.7	1.16 (0.73–1.86)
British Columbia	11.9	65.4	31.1	1.99 (1.59–2.49)	3.5	0.89 (0.52–1.51)
Territories	0.5	75.6	14.6	0.81 (0.62–1.06)	9.8	2.17 (1.47–3.22)
Urban/Rural						
Rural	17.8	71.3	24.6	0.83 (0.70–0.98)	4.2	0.81 (0.57–1.15)
Urban (< 30,000)	17.0	70.2	26.4	0.91 (0.77–1.08)	3.4	0.66 (0.45–0.97)
Urban (30,000-99,000)	8.4	79.8	26.6	0.92 (0.73–1.15)	3.6	0.71 (0.42–1.21)
Urban (100,000-499,999)	11.6	65.4	29.9	1.10 (0.91–1.33)	4.7	0.99 (0.65–1.50)
Urban (500,000+)	45.1	67.3	27.9	1.00	4.9	1.00
Travelled to another city or town for birth						
Did not travel	75.3	68.3	27.3	1.00	4.4	1.00
< 80 km	20.7	68.7	27.2	0.99 (0.86–1.15)	4.2	0.93 (0.68–1.27)
80 km+	4.0	67.7	30.2	1.12 (0.84–1.48)	2.1	0.48 (0.24–0.94)
Education						
Highschool or less	21.0	67.0	25.7	1.00	7.3	1.00
Post-secondary, below bachelor	43.6	68.6	27.2	1.03 (0.88–1.22)	4.1	0.55 (0.41–0.74)
Bachelor	25.7	69.6	27.7	1.04 (0.87–1.24)	2.7	0.35 (0.24–0.52)
Graduate	9.8	66.9	30.5	1.19 (0.94–1.50)	2.6	0.36 (0.20–0.66)
Employment during pregnancy						
Yes	78.7	69.0	27.4	0.98 (0.84–1.14)	3.6	0.48 (0.37–0.63)
No	21.3	66.2	26.8	1.00	7.1	1.00
Household income (Canadian Dollar)						
< 30,000	17.0	68.0	25.7	1.00	6.2	1.00
30,000- < 60,000	30.7	68.6	25.3	0.98 (0.81–1.17)	6.0	0.96 (0.70–1.32)
60,000- < 100,000	32.2	72.6	24.8	0.90 (0.75–1.08)	2.6	0.39 (0.27–0.58)
100,000+	20.1	64.4	33.5	1.38 (1.12–1.69)	2.1	0.35 (0.21–0.58)

^a^Obtained using bivariable multinomial logistic regression models using ‘Appropriate’ as the reference category

^b^Other includes: Nurse or nurse practitioner, other doctor (unspecified) or a response of ‘other’ to the question asking about the type healthcare provider that provided most of the respondent’s prenatal care

The maternal factors that were significantly associated with a higher likelihood of early first prenatal ultrasound were: fertility medications or procedures (adjusted OR = 3.47, 95%CI: 2.59–4.65), health problems before pregnancy (adjusted OR = 1.30, 95%CI: 1.09–1.56), health problems during pregnancy (adjusted OR = 1.27, 95%CI: 1.09–1.48) and underweight BMI (adjusted OR = 1.81, 95%CI:1.34–2.44) (Table 2). As for the ‘late’ category, women who had health problems before pregnancy were significantly less likely to have a late first prenatal ultrasound than those who did not have those problems (adjusted OR = 0.55, 95%CI: 0.31–0.97) (Table 2). In addition, women whose pregnancies were unintended were significantly more likely to undergo late first ultrasound than those with intended pregnancies (adjusted OR = 1.89, 95%CI: 1.38–2.59) (Table 2). No other maternal factors were significantly associated with late first prenatal ultrasound.

**Table 2:** Adjusted Odds Ratios (OR) and 95% Confidence Intervals (95%CI) obtained from a multivariable multinomial logistic regression model for timing of ultrasound at different independent variables. The Maternity Experiences Survey (MES)

Variable	Early Adjusted OR (95%CI)^a^	Late Adjusted OR (95%CI)^a^
MATERNAL FACTORS		
Fertility medications or procedures		
Yes	3.47 (2.59–4.65)	1.39 (0.33–5.80)
Health problems before pregnancy		
Yes	1.30 (1.09–1.56)	0.55 (0.31–0.97)
Health Problems during pregnancy		
Yes	1.27 (1.09–1.48)	1.05 (0.73–1.50)
Pre-pregnancy BMI		
Underweight (less than 18.5)	1.81 (1.34–2.44)	0.87 (0.39–1.93)
Overweight/obese (25+)	1.06 (0.92–1.23)	0.99 (0.70–1.39)
Intended pregnancy		
Unintended	1.13 (0.96–1.34)	1.89 (1.38–2.59)
BEHAVIOURAL RISK FACTORS		
Smoking during the last 3 months of pregnancy		
Yes	0.97 (0.76–1.24)	1.00 (0.62–1.61)
Alcohol during pregnancy		
Yes	0.69 (0.53–0.90)	1.01 (0.57–1.79)
REPRODUCTIVE HISTORY		
Parity		
Multiparous	0.67 (0.57–0.78)	1.41 (0.97–2.05)
History of premature birth		
Yes	1.41 (1.06–1.89)	0.56 (0.24–1.32)
History of ectopic pregnancy or stillbirth		
Yes	1.50 (1.00–2.26)	0.40 (0.14–1.19)
History of miscarriage		
Yes	2.04 (1.65–2.54)	0.61 (0.33–1.11)
History of therapeutic abortion		
Yes	1.01 (0.81–1.25)	0.89 (0.54–1.47)
PRENATAL AND BIRTH RELATED FACTORS		
Type of prenatal care provider		
Family doctor/GP	0.94 (0.78–1.12)	1.26 (0.89–1.77)
Midwife and other^b^	1.16 (0.86–1.57)	1.71 (0.94–3.11)
Mode of delivery		
Caesarean	1.11 (0.95–1.29)	1.14 (0.81–1.62)
Birthweight		
< 2500	1.25 (0.89–1.77)	0.76 (0.28–2.09)
> 4000	0.90 (0.73–1.13)	0.96 (0.59–1.58)
SOCIO-DEMOGRAPHIC FACTORS		
Maternal age at delivery		
< 20	0.54 (0.34–0.84)	1.11 (0.53–2.33)
35+	0.92 (0.76–1.12)	0.93 (0.61–1.42)
Country of birth/Aboriginal status		
Born outside of Canada	0.82 (0.67–0.99)	1.75 (1.14–2.68)
Aboriginal	0.83 (0.58–1.19)	0.59 (0.28–1.24)
Marital status		
With partner	0.80 (0.60–1.08)	0.92 (0.51–1.65)
Province of prenatal care		
Newfoundland and Labrador	1.66 (1.20–2.30)	1.25 (0.54–2.86)
Prince Edward Island	0.84 (0.60–1.18)	1.60 (0.85–3.01)
Nova Scotia	1.68 (1.25–2.28)	1.24 (0.57–2.70)
New Brunswick	1.29 (0.90–1.87)	1.14 (0.57–2.29)
Ontario	2.16 (1.76–2.65)	0.98 (0.63–1.54)
Manitoba	0.66 (0.45–0.98)	2.88 (1.64–5.05)
Saskatchewan	1.50 (1.05–2.14)	1.12 (0.53–2.36)
Alberta	1.37 (1.05–1.77)	1.55 (0.87–2.78)
British Columbia	1.90 (1.45–2.50)	0.84 (0.44–1.58)
Territories	1.01 (0.68–1.52)	4.50 (2.27–8.93)
Urban/Rural		
Rural	0.83 (0.66–1.03)	0.94 (0.57–1.57)
Urban (< 30,000)	0.87 (0.70–1.07)	0.58 (0.35–0.98)
Urban (30,000-99,000)	0.94 (0.72–1.24)	0.93 (0.51–1.70)
Urban (100,000-499,999)	0.90 (0.71–1.13)	1.21 (0.69–2.14)
Travelled to another city or town for birth		
< 80 km	0.99 (0.83–1.19)	1.28 (0.88–1.86)
80 km+	1.24 (0.87–1.75)	0.28 (0.12–0.65)
Education		
Post-secondary, below bachelor	0.96 (0.78–1.19)	0.74 (0.51–1.07)
Bachelor	0.98 (0.77–1.25)	0.48 (0.29–0.80)
Graduate	0.91 (0.68–1.22)	0.55 (0.27–1.10)
Employment during pregnancy		
Yes	0.96 (0.79–1.17)	0.83 (0.57–1.21)
Household income (Canadian Dollar)		
30,000- < 60,000	1.02 (0.81–1.28)	1.37 (0.92–2.05)
60,000- < 100,000	0.94 (0.74–1.21)	0.74 (0.45–1.24)
100,000+	1.41 (1.07–1.85)	0.74 (0.38–1.47)
INTERACTION: Prenatal care provider x History of miscarriage		
OB/GYN x No miscarriage	1.00	1.00
Family doctor x miscarriage	0.90 (0.64–1.25)	0.76 (0.29–1.98)
Midwife and other x miscarriage	0.31 (0.14–0.66)	1.51 (0.20–11.35)

^a^Obtained using a multivariable multinomial logistic regression model using ‘Optimal’ as the reference category

^b^Other includes: Nurse or nurse practitioner, other doctor (unspecified) or a response of ‘other’ to the question asking about the type healthcare provider that provided most of the respondent’s prenatal care

In terms of the behavioural risk factors, women who used alcohol during pregnancy were significantly less likely to receive early first prenatal ultrasounds than those who did not use alcohol (adjusted OR = 0.69, 95%CI: 0.53–0.90) (Table 2). No other behavioural risk factors were significantly associated with early or late first prenatal ultrasound.

As for reproductive history factors, history of premature birth was significantly associated with an increased likelihood of early first prenatal ultrasound (adjusted OR = 1.41, 95%CI: 1.06–1.89) (Table 2). Being multiparous was associated with a decreased likelihood of early first prenatal ultrasound (adjusted OR = 0.67, 95%CI: 0.57–0.78) (Table 2). The only interaction term that was found to be significant was that between prenatal care provider and whether the woman had a history of miscarriage. Taking this interaction term into account, having a history of miscarriage while seeing an OB/GYN (the reference category for care provider) was significantly associated with early first prenatal ultrasound (adjusted OR = 2.04, 95%CI: 1.65–2.54), whereas having a prenatal care provider other than a family doctor or an OB/GYN combined with having a history of miscarriage had significantly lower OR for early first prenatal ultrasound as compared to those who have had a history of miscarriage and were seeing an OB/GYN (adjusted OR = 0.31, 95%CI: 0.14–0.66) (Table 2). No other reproductive history factors were significantly associated with early or late first prenatal ultrasound.

The socio-demographic factors that were significantly associated with increased likelihood of early first prenatal ultrasound were a household income of 100,000 Canadian Dollars or more (adjusted OR = 1.41, 95%CI: 1.07–1.85) and province of prenatal care (Table 2). Women who received prenatal care in Newfoundland and Labrador (adjusted OR = 1.66, 95%CI: 1.20–1.30), Nova Scotia (adjusted OR = 1.68, 95%CI: 1.25–2.28), Ontario (adjusted OR = 2.16, 95%CI: 1.76–2.65), Saskatchewan (adjusted OR = 1.50, 95%CI: 1.05–2.14), Alberta (adjusted OR = 1.37, 95%CI: 1.05–1.77) and British Columbia (adjusted OR = 1.90, 95%CI: 1.45–2.50) were significantly more likely to receive early first prenatal ultrasound than those receiving their care in Quebec (Table 2). On the other hand, women who received their prenatal care in Manitoba were significantly less likely to receive early prenatal ultrasound (adjusted OR = 0.66, 95%CI: 0.45–0.98). The factors associated with a lower likelihood of early first prenatal ultrasound were being born outside of Canada (adjusted OR = 0.82, 95%CI: 0.67–0.99) and being under 20 years of age at the time of birth (adjusted OR = 0.54, 95%CI: 0.34–0.84) when compared to being Canadian born and being between 20 and 34 years of age at the time of birth, respectively. Being born outside of Canada was also significantly associated with a higher likelihood of late first ultrasound than those born in Canada (adjusted OR = 1.75, 95%CI: 1.14–2.68) as was receiving prenatal care in Manitoba and the Territories (adjusted OR = 2.88, 95%CI: 1.64–5.05 and adjusted OR = 4.50, 95%CI: 2.27–8.93, respectively) than those receiving their care in Quebec (Table 2). The factors that were significantly associated with a lower likelihood of late first prenatal ultrasound were: living in an urban setting with a population size less than 30,000 (adjusted OR = 0.58, 95%CI: 0.35–0.98) when compared to urban population size of 500,000+, having a bachelor’s degree (adjusted OR = 0.48, 95%CI: 0.29–0.80) when compared to women who had a highschool education or less, and having travelled 80 km or more to give birth (adjusted OR = 0.28, 95%CI: 0.12–0.65) when compared to women who did not travel for birth (Table 2).

## Discussion

The results of this study indicate that only about 68% of Canadian women received an optimally timed prenatal ultrasound. Around 27% of Canadian women received early ultrasounds and this was influenced by a number of factors including: being younger, underweight, born outside of Canada, having a high household income and receiving prenatal care in Newfoundland and Labrador, Nova Scotia, Ontario, Saskatchewan, Alberta or British Columbia when compared with residing in Quebec. On the other hand, late ultrasound was performed in around 4% of Canadian women and was influenced by: being born outside of Canada, receiving prenatal care in Manitoba or the Territories and having an unintended pregnancy. The results of this study can be used to better direct national educational efforts about the optimal timing of the first prenatal ultrasound in low risk pregnancy. In addition, these results may be of use when addressing large scale issues such as over- and under-utilization of ultrasound in pregnancy.

Based on the present study, only 68% of Canadian women received optimally timed prenatal ultrasounds. This is concerning because both over- and under-utilization of prenatal ultrasound can be problematic. The obvious consequences of overutilization lie in the cost associated with performing these tests, which can burden the healthcare system if used excessively [36], while underutilization may lead to missed opportunities for screening [19].

This study has found that, after adjusting for confounders, multiple maternal factors were associated with a higher likelihood of early but not late first prenatal ultrasound. Women who used fertility procedures or medications for this pregnancy were significantly more likely to have an early prenatal ultrasound. These women generally undergo more testing than other women do and require multiple ultrasounds in early pregnancy to check embryonic growth and development [37]. Similarly, women who had pre-pregnancy or pregnancy conditions that warranted additional care during pregnancy may require earlier first prenatal ultrasounds than other women. In addition, underweight women were significantly more likely to have an early ultrasound. Obese women are more likely to access prenatal care late in pregnancy than non-obese women [19] and are generally more likely to avoid or delay screening tests [38, 39] due to multiple factors including negative body image and to avoid weight loss advice [39], while underweight women are less likely to have late access to prenatal care [19]. In addition to this, underweight women have a higher likelihood of irregular menstruation [40] which may result in uncertain dates and, consequently, an earlier first prenatal ultrasound to establish gestational age. The present study also found that unintended pregnancy was significantly associated with increased likelihood of late ultrasound. This is in agreement with previous findings that women who have unintended pregnancies tend to have delayed prenatal care [41] and possibly miss the window of an optimally timed prenatal ultrasound.

After adjusting for covariates, the present study also found that women who used alcohol during pregnancy were less likely to undergo early first prenatal ultrasound than those who did not. This is consistent with previous studies that have found that substance use, including alcohol use, was associated with late access to prenatal care [22, 23, 42, 43], which consequently makes them less likely to have early ultrasounds as opposed to optimal ultrasounds. A possible explanation of this is that these women may believe that their substance abuse has already harmed their baby irreversibly, leading them to delay prenatal care [44]. Another possible explanation, though no previous studies were found to support this, is that these women might not realize that they are pregnant early enough, leading to delayed prenatal care.

The present study found that reproductive history factors are significantly associated with timing of the first prenatal ultrasound, after adjusting for confounders. Multiparous women were less likely to receive early ultrasounds than primiparous women. This is consistent with previous studies in multiple countries including Canada, that have found that higher parity is associated with late or inadequate access to prenatal care [20, 21, 23, 45, 46]. Women who had positive previous pregnancies may feel more confident than women who are pregnant for the first time, and may not feel that accessing prenatal care early is of value [47, 48]. On the other hand, women who have had negative experiences in their previous pregnancy may want to avoid or delay prenatal care [47]. Moreover, since these women most likely have children already, they may struggle with child care issues and time constraints leading to delayed prenatal care [47, 48], and, consequently, leading to a lower likelihood of having early ultrasounds as opposed to optimally timed ones. The present study also found that women who had a history of preterm birth were significantly more likely to undergo early prenatal ultrasound. Although no specific recommendations have yet been set by the SOGC about the optimal timing of cervical length measurement using ultrasound [49], these women and their healthcare providers may opt for earlier ultrasounds to measure the cervix in hopes of avoiding preterm birth in this pregnancy. Interestingly, the present study also found an interaction effect between type of prenatal provider and having a history of a miscarriage. Specifically, women who received most of their care from and OB/GYN and had a history of miscarriage were at higher odds of receiving an early first ultrasound. In addition, among patients who had a history of miscarriage, women who were seeing healthcare providers other than OB/GYN or family doctor had significantly lower ORs for early ultrasound than those who received care from an OB/GYN. Midwives may be less reliant on ultrasound while caring for a patient [50], and may therefore not treat patients with a history of miscarriage with as many diagnostic tests as OB/GYNs do. In addition, in theory, women who had a history of a miscarriage may prefer to receive their care from an OB/GYN or family doctor, due to the fact that having a history of miscarriage may predispose women to a subsequent miscarriage [51]. This may mean that other healthcare providers may not see as many patients with a history of miscarriage as OB/GYN or family doctor. It is important to note here that it is not a specific recommendation of the SOGC that women with a history of miscarriage have earlier ultrasounds during subsequent pregnancies. However, these women and their prenatal care providers may be more anxious about the well being of the baby and may, therefore, opt for earlier ultrasounds.

In terms of socio-demographic variables, the present study also found that women who were under 20 years of age were less likely to have an early ultrasound. This is consistent with findings that women who are under 20 years of age are more likely to have late access to prenatal care [19, 52], which can reduce their likelihood of receiving earlier ultrasound compared to optimally timed ones. Teenage mothers may delay access to prenatal care due to not realising that they are pregnant [53], or due to fears of confirming that they are pregnant or the fear that someone else might subsequently discover their pregnancy [54]. Another finding of the present study is that women born outside of Canada were less likely to receive an early ultrasound and more likely to receive a late ultrasound. Previous studies in multiple countries including Canada have reported that foreign born mothers were more likely to have late access to prenatal care than mothers who were born in the country of reference [22, 55–57]. This may be due to language barriers, fear of discrimination and lack of knowledge of the local healthcare system [58, 59]. The present study also found differences between provinces with respect to timing of ultrasound. Patients receiving their prenatal care in Newfoundland and Labrador, Nova Scotia, Ontario, Saskatchewan, Alberta and British Columbia were more likely to receive early ultrasounds than those receiving their care in Quebec. The present study also found that receiving prenatal care in Manitoba was not only associated with a lower likelihood of early ultrasound but also with a higher likelihood of late ultrasound. These findings may be explained by the differences in prevalence of inadequate prenatal care between provinces; according to the MES, Quebec has a higher prevalence of inadequate prenatal care than multiple provinces including Newfoundland and Labrador, Nova Scotia, Ontario, Saskatchewan, Alberta and British Columbia [22]. Quebec also has a lower prevalence of inadequate prenatal care than 2 of the Territories and Manitoba, although the difference between Quebec and Manitoba was small (22.3 and 22.5%, respectively) [22]. Moreover, women who receive their prenatal care in Ontario generally receive more ultrasounds during pregnancy than those in other provinces [50], which is consistent with the finding of the present study that the highest prevalence of early ultrasound was found in Ontario. Another factor contributing to these provincial differences can be the differences in wait times for ultrasound examinations in the Canadian provinces. In 2005 and 2006, Manitoba had higher ultrasound wait times than most of the other provinces followed by Quebec [60]. In 2006, it was reported that in Manitoba the wait time for an ultrasound was 8 weeks, followed by Quebec and Nova Scotia, (each reporting 6 week median wait times), with the other provinces reporting median wait times of 2–4.8 weeks [60]. As of 2018, the shortest wait time for an ultrasound was reported in Saskatchewan (1.1 weeks) and the longest was reported in Newfoundland and Labrador (10.5 weeks) [61]. Interestingly, some of the shortest wait times for an ultrasound were consistently found in Ontario (2 weeks) [60, 61]. The present study also found that receiving prenatal care in the Territories was strongly associated with a higher likelihood of late prenatal ultrasound. The effect of wait times may be extrapolated to the Territories where access to prenatal care and diagnostic technology may be reduced. Another finding of the present study was that women living in an urban setting of a population of 30,000 or less and women who had to travel 80 km or more to give birth were less likely to receive late ultrasounds. Although no specific findings from previous literature were found to support this, one explanation can be that in smaller settings, where large hospitals and centers are not available to provide all services, patients may have closer, more familiar relationships with their prenatal care providers making them more likely to access prenatal care and diagnostic testing at an optimal time. Education was a factor that influenced late ultrasound but not early ultrasound in the present study, where having a bachelor’s degree was associated with a lower likelihood of having a late ultrasound when compared to highschool education or less. Women with higher education tend to have more adequate access to prenatal care [46, 52]. However, pregnant women with very high education may have more developed critical thinking skills allowing them to be more comfortable to question the practices of healthcare providers [46, 62] and, therefore, may not be inclined to undergo testing as early as recommended. The present study also found that women who reported having a household income of 100,000 Canadian Dollars or more were more likely to receive early prenatal ultrasound. This is a surprising finding since medically necessary health care services are free to residents of Canada [63], making it less likely for Canadians to have financial barriers preventing access to prenatal care. However, this effect can be explained by taking into consideration the environmental and personal factors that influence women of different income brackets. The socio-ecological model of determinants of health services utilization proposed by Sword suggests that women of differing income brackets can have different personal, environmental and political influencers, ultimately leading to differing health services utilization patterns [64]. These differences can play a role even within a universally funded healthcare system and may make Canadian women of higher income more likely to prioritize their prenatal care when compared to lower income women, making them more likely to ask for and receive additional prenatal testing to ‘make sure the baby is ok’. Moreover, higher income Canadian women may have more flexibility to take ‘time off’ to go to prenatal appointments than lower income women do, making them more likely to access prenatal services early in pregnancy.

The main limitation of this study is the cross-sectional design of the MES which may lead to reverse causality. In addition, the MES is over a decade old and the current Canadian population may have different characteristics than those captured by the MES. However, even though the MES is ‘old’, the information from this study can be valuable to establish a baseline of data pertaining to timing of prenatal ultrasound for future hypothesis generation. In addition, ‘old’ data can be valuable because, should there be newer data in the future, the results of this study can be used to investigate trends and changes over time in Canada. It is also important to note that the recommendations for prenatal ultrasound have not changed much since the time of the survey [10, 31–33]. Another limitation is that with self-reported data there is always a potential for information bias either due to lack of recall or the temptation to present oneself favourably. Finally, not all of the possible confounding variables such as irregular menstrual cycles can be adjusted for either due to the lack of their availability in the MES or due to the power limitations within the regression model. Despite all the limitations mentioned above, the MES is the largest, most up to date Canada-wide database that covers information about timing of ultrasound and different aspects of the maternity experience, in addition to having a high response rate of 78%.

## Conclusions

The findings of this study indicate only 68% of Canadian women receive an optimally timed prenatal ultrasound, and that the timing of prenatal ultrasound is influenced by numerous factors such as province of prenatal care, maternal age and country of birth. This study also found that having a history of a miscarriage combined with having a prenatal care provider other than an OB/GYN or family doctor was associated with a lower likelihood of early ultrasound. These findings establish a baseline of factors influencing the timing of prenatal ultrasound in Canada that can be built upon by future studies, which can investigate the relationship between type of prenatal care provider and province of prenatal care in a more up to date context, and perhaps focusing on provincial settings. In addition, these findings can help guide efforts to encourage the use of optimally timed prenatal ultrasounds by focusing education based on province, prenatal care provider, and patient characteristics. This will potentially address issues including under- and over-utilization of ultrasound in pregnancy.

## Abbreviations

95%CI: 95% Confidence Interval
BMI: Body Mass Index
MES: Maternity Experiences Survey
OR: Odds Ratio
RDC: Research Data Centre
